# New species of *Vomerina* Winterton (Diptera, Therevidae, Agapophytinae) from Australia

**DOI:** 10.3897/zookeys.218.2380

**Published:** 2012-08-30

**Authors:** Shaun L. Winterton, David J. Ferguson

**Affiliations:** 1 California State Collection of Arthropods, California Department of Food & Agriculture, Sacramento, California, USA; 2 Australian National Insect Collection, CSIRO Ecosystem Sciences, Black Mountain, Canberra, Australia

**Keywords:** Asiloidea, Therevidae, Australia

## Abstract

Two new species of *Vomerina* Winterton, 2007 (*Vomerina comapenis* **sp. n.** and *Vomerina micora* **sp. n.**) are described from New South Wales (Australia).

## Introduction

The endemic Australasian stiletto fly (Diptera: Therevidae) fauna is exclusively placed in two subfamilies, Agapophytinae and Therevinae (Winterton 2009, 2011). A key to genera of the region can be found in Winterton (2011). Therevinae are a diverse, cosmopolitan subfamily while Agapophytinae are endemic to Australasia and South America. Agapophytinae comprise 197 species in 26 genera; 23 genera in Australasia and three genera (*Entesia* Oldroyd, 1968, *Melanothereva* Malloch, 1932 and *Pachyrrhiza* Philippi, 1865) in Argentina and Chile (Winterton 2006). *Vomerina* Winterton, 2007 is a previously monotypic Australian genus of distinctively black stiletto flies with a matte white to silver pleural stripe. The genus can be differentiated from all other Agapophytinae by this coloration, and by the male gonocoxites having a large plow-shaped ventral lobe and lacking a medial atrium. *Vomerina* is the putative sister genus to *Bonjeania* Irwin & Lyneborg, 1989, another endemic Australian genus of 18 described species (Winterton 2007). Two new species of *Vomerina* (*Vomerina comapenis* sp. n. and *Vomerina micora* sp. n.) are described and figured herein from New South Wales (Australia). A revised diagnosis of *Vomerina* and a key to species are also presented.

## Material and methods

Adult morphological terminology follows McAlpine (1981) as modified by Winterton et al. (1999a) and Winterton (2006). Genitalia were macerated in 10% KOH at room temperature for one day to remove soft tissue, then rinsed in distilled water and dilute acetic acid, and dissected in 80% ethanol. Preparations were then placed into glycerine, with images made with the aid of a digital camera. Specimen images at different focal points were taken using a digital camera and subsequently combined into a serial montage image using Helicon Focus (©HeliconSoft). Genitalia preparations were placed in glycerine in a genitalia vial mounted on the pin beneath the specimen. The following collection acronyms are cited in the text: Australian National Insect Collection (Canberra) (ANIC), Australian Museum (AM).

## Taxonomy

### Vomerina

Winterton, 2007

urn:lsid:zoobank.org:act:7781FCA5-2F6F-49CF-B629-1CAB9799A914

http://species-id.net/wiki/Vomerina

#### Type species.

*Vomerina humbug* Winterton, 2007: 22.

#### Diagnosis.

Body length 6.5–8.0 mm. Body glossy black with matte white (or silver) lateral stripe of dense pubescence on pleuron, usually extending to anterior segments of abdomen; male frons wider than ocellar tubercle at narrowest point; parafacial setae absent; male with single row of postocular setae; lower frons and face slightly to greatly protruding anteriorly; antenna length shorter than or equal to head length; scape cylindrical or bulbous; flagellum conical, style terminal; sternum with medial furrow lacking setal pile; posterior surface of mid coxa lacking setal pile; wing cell *m_3_* closed; elongate velutum patches present on fore and hind femora; femora without macrosetae; male genitalia without medial atrium, inner gonocoxal process absent or greatly reduced; ventral lobe large, plowshare-shaped, projecting posteromedially with dense medial covering of velutum; dorsal apodeme of parameral sheath well sclerotized; ejaculatory apodeme and lateral ejaculatory apodeme short; gonocoxal apodeme much shorter than gonocoxite length; female with three spermathecae; spermathecal ducts joined to common spermathecal sac duct; acanthophorite spines A1 and A2 present, well developed.

#### Comments.

Winterton (2007) erected *Vomerina* based on the male of *Vomerina humbug*. The stripe of pubescence on the lower portion of the pleuron is distinctively matte white (almost glaucous gray) in *Vomerina humbug* and *Vomerina comapenis* sp. n., where it continues onto the posterior surface of the head and onto the anterior segments of the abdomen. In *Vomerina micora* sp. n., the stripe of dense pubescence is present only on the thorax and is silver. This type of pleural stripe is also found in many species of *Bonjeania* (e.g. *Bonjeania zwicki* Winterton, 2007) and numerous species of *Parapsilocephala* Kröber, 1912 and *Acraspisa* Kröber, 1912. There is a distinct similarity in body shape between *Vomerina* and certain *Bonjeania* species such as *Bonjeania webbi* Winterton 2007 and *Bonjeania bapsis* Winterton, 2007. All have a conical head and similar body shape, but most *Bonjeania* species lack the distinct pleural stripe of pubescence, have a medial atrium and small ventral lobe on the male gonocoxites, while females have only a single spermatheca.

#### Included species.

*Vomerina comapenis* sp. n., *Vomerina humbug* Winterton, 2007, *Vomerina micora* sp. n.

#### Key to *Vomerina* species

**Table:** 

1	Wing with costal area slightly infuscate, rest of wing hyaline; frons strongly projecting anteriorly; scape longer than flagellum, bulbous; pale setae on katatergite	*Vomerina humbug* Winterton, 2007
–	Wing infuscation more extensive; frons projecting only slightly; scape shorter than flagellum, cylindrical, not bulbous; dark setae on katatergite	2
2	Matte white lateral pubescence extending from pleuron onto anterior segments of abdomen as a continuous stripe; hind tibia with yellow band midway	*Vomerina comapenis* sp. n. (Figs 1–2)
–	Silver lateral pubescence on pleuron only, absent from abdomen; hind tibia without yellow band	*Vomerina micora* sp. n. (Figs 5–7)

### Vomerina comapenis sp. n.

urn:lsid:zoobank.org:act:9249B712-FFB3-47A7-A34F-89EC0ECE7461

http://species-id.net/wiki/Vomerina_comapenis

**Figure 1.**
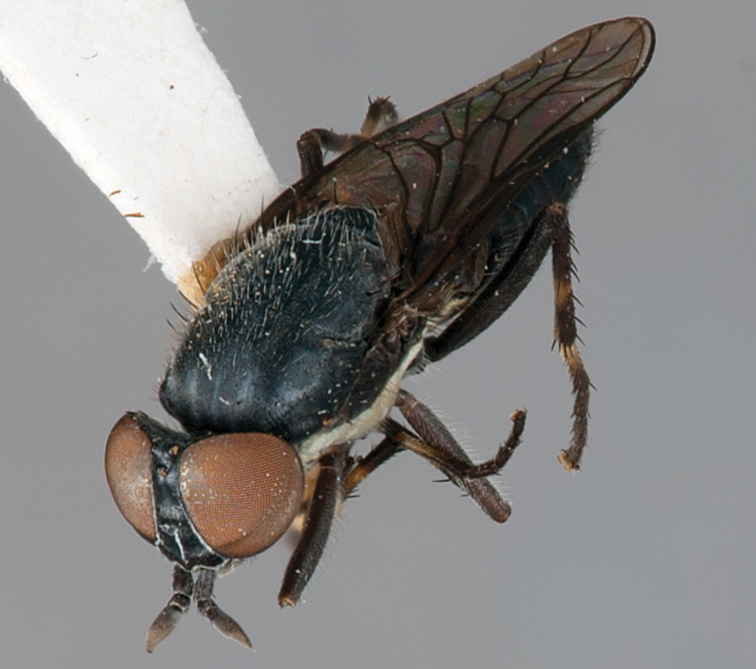
*Vomerina comapenis* sp. n., male habitus, oblique view. Body length = 7.0 mm.

Figs 1 [Fig F2] [Fig F3] –4

#### Type material.

– **Holotype** male, AUSTRALIA: **New South Wales:** Boonanghi State Forest, 24 km W Kempsey, vic.[inity of] ‘The Blowhole’ [-31.0747, 152.5663], 8.x.1993, G. & A. Williams, riparian zone, dry rainforest (AM).

#### Diagnosis.

Wing dark infuscate; frons slightly projecting around antennal base; scape cylindrical, shorter than flagellum, not bulbous; black setae on katatergite; white pubescence on pleuron extending onto abdomen; male abdomen black apically; articulated inner gonocoxal process present, greatly reduced.

#### Description.

Body length: 7.0 mm. *Head*. Frons flat, surface rugose-striated medially, wider than ocellar tubercle at narrowest point, antennal base positioned low on frons; lower frons and face slightly protruding around antennal base; frons glossy black, silver-grey pubescence along eye margin; short setae sparsely distributed on lower frons; narrow medial stripe of silver pubescence around antennal bases on face, parafacial and along margin of eye; ocellar tubercle flat, glossy black; occiput concave, black, overlain with dense grey pubescence; single row of very short, black postocular setae; gena black, overlain with white pubescence admixed with white, elongate setae; palpus and labellum brown-black with sparse, dark setae; antenna black, only slightly shorter than head; short dark setae on scape and pedicel; scape shorter than flagellum length, cylindrical, with grey pubescence; flagellum conical with brownish suffusion. *Thorax*. Black, scutum and scutellum overlain with grey-black pubescence admixed with relatively short, dark setae; scutum with narrow, faint dorsocentral and medial stripe of pale grey pubescence; scutal macrosetae black; pleuron, sternum and coxae glossy black; distinctive broad stripe of matte white to silver pubescence along pleuron length; elongate pale setae on proepisternum, katepisternum and coxae, black setae on anepisternum and katatergite; legs black with pale setae of various lengths on femora, dark yellow band midway on mid and hind tibiae and basitarsus; wing smoky infuscate; venation dark; haltere brown-black; scutal chaetotaxy (pairs): notopleural, 4; supra alar, 1; post alar, 1; dorsocentral, 1–2; scutellar, 1. *Abdomen*. Glossy black, uniform scattered pale setae, longer laterally, white-grey pubescence laterally on sternites 1–3. *Male genitalia*. Gonocoxite with outer gonocoxal processes reduced; articulated inner gonocoxal process greatly reduced; gonostylus broad; ventral lobe large, plowshare-shaped, projecting posteroventrally with velutinous pubescence on ventral surface (Figs 3–4).

**Figure 2. F2:**
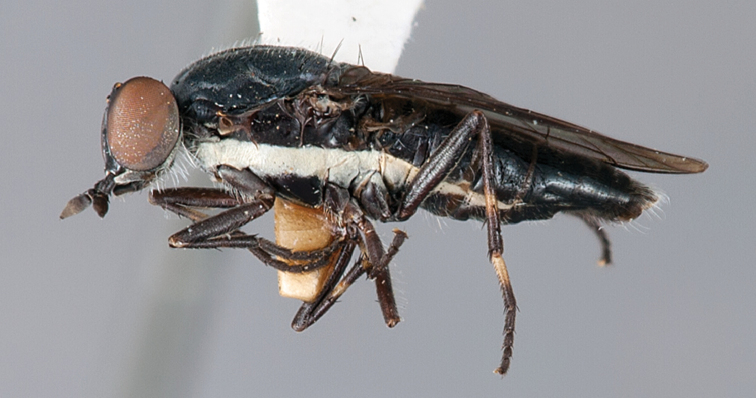
*Vomerina comapenis* sp. n., male habitus, lateral view. Body length = 7.0 mm.

**Figure 3. F3:**
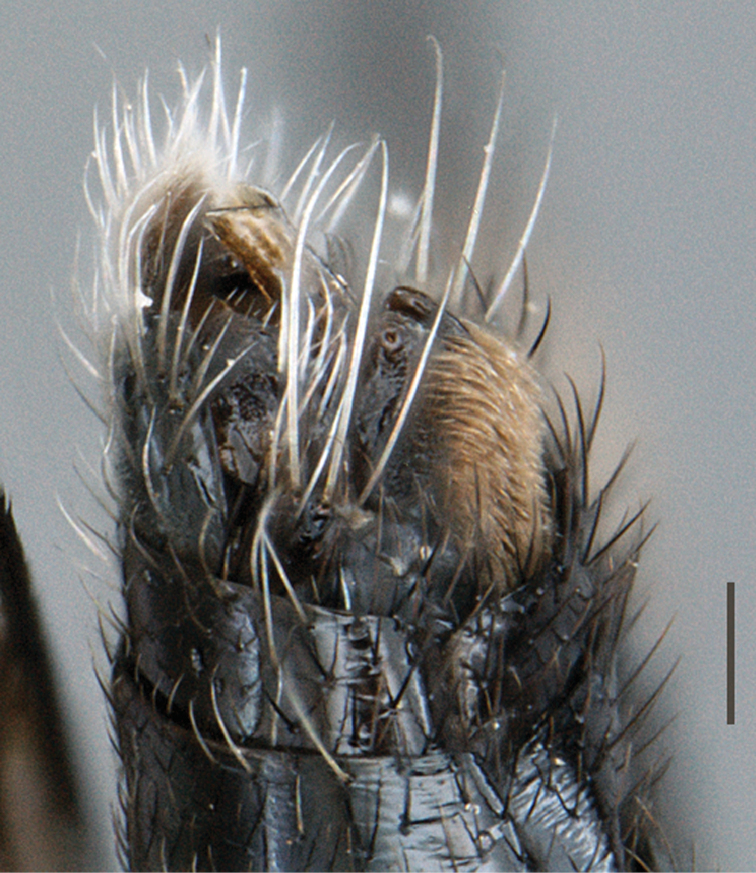
*Vomerina comapenis* sp. n., male terminalia, lateral view. Scale line = 0.2 mm.

**Figure 4.**
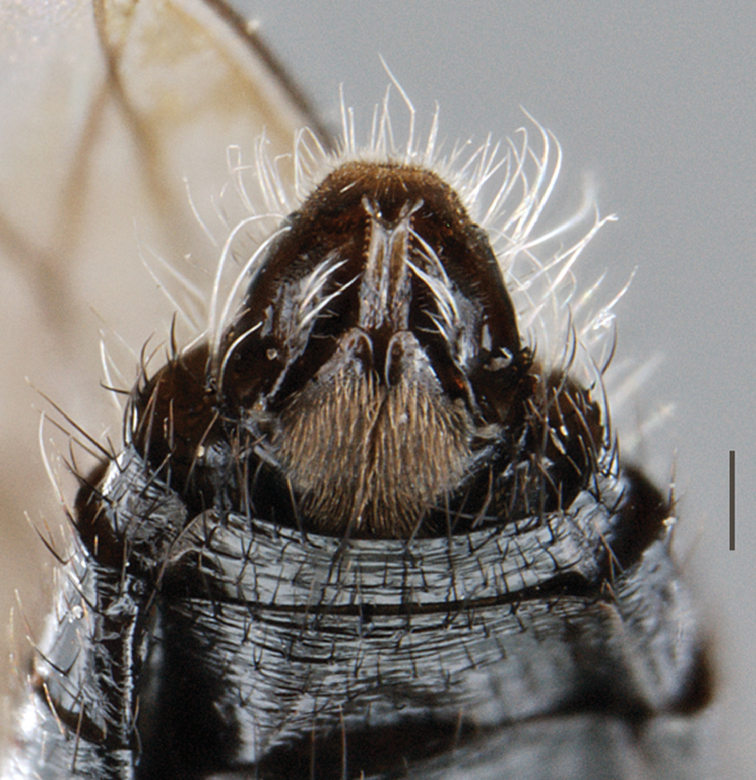
*Vomerina comapenis* sp. n., male terminalia, ventral view. Scale line = 0.2 mm.

#### Etymology.

The species epithet is derived from the Latin, *coma* hairy; *penis*, intromitant organ; referring to the vestiture of the male genitalia.

#### Comments.

*Vomerina comapenis* sp. n. is muchsmaller in body size to *Vomerina humbug* and can be further distinguished by the frons being only slightly projecting, a non-bulbous antennal scape and darker wings. This species is distinguished from *Vomerina micora* sp. n. by the lateral velutum stripe extending onto the abdomen, abdomen uniformly black, and the scutum lacking grey pubescent dorsocentral stripes. The female is unknown.

### Vomerina micora sp. n.

urn:lsid:zoobank.org:act:79F15746-5D56-4BB6-8D2A-4CF20E68AC0F

http://species-id.net/wiki/Vomerina_micora

Figs 5 [Fig F6] –7

#### Type material.

**– Holotype** female, AUSTRALIA: **New South Wales:** South Black Range, 8 km E Hoskinstown, Fluoro U.V. light trap [-35.4183, 149.5347], 11.ii.2010, 1180m D. J. Ferguson (ANIC 29 029246)

#### Diagnosis.

Wing dark infuscate; scape cylindrical, shorter than flagellum; black setae on katatergite; silver pubescence on pleuron not extending onto abdomen; scutum with two faint dorsocentral stripes bordering a darker medial stripe; female abdominal segments 7–8 orange.

#### Description.

Body length: 6.5 mm. *Head*. Frons flat, rugose-striated medially, wider than ocellar tubercle at narrowest point, antennal base positioned low on frons; lower frons and face protruding only slightly with curved ridge above antennal base; frons glossy black, short setae sparsely distributed on upper frons, silver-grey pubescence on parafacial, oral cavity and along eye margin; ocellar tubercle flat, black; occiput concave, black, overlain with grey pubescence; few relatively strong short black postocular setae followed by weaker setae in several irregular rows; elongate black setae along lower postocular admixed with white setae along gena; gena black, overlain with silver-grey pubescence; palpus and labellum brown-black with sparse, dark setae; antenna two-thirds length of head; scape and pedicel black with short dark setae; scape about half length of flagellum, cylindrical, with grey pubescence; flagellum with red-brownish suffusion and grey pubescence. *Thorax*. Glossy black; scutum and scutellum overlain with grey-black pubescence admixed with relatively short, dark setae; scutum with pair of narrow dorsocentral stripes of sparse pale-grey pubescence bordering a darker medial stripe; scutal macrosetae black; pleuron and sternum glossy black; broad stripe of silver pubescence along pleuron from proepisternum to hind coxa; fore and middle coxae dark brown, hind coxa black; posterior surface of hind coxa with silver pubescence; elongate pale setae on proepisternum and coxae, black setae on katatergite; fore and middle femora dark brown, hind femur black; elongate yellowish-grey velutum patches to ventral surface of hind and apical half of fore femora; pale setae of various lengths on all femora; tibiae black to dark brown; mid and hind basitarsi dark yellow basally; wing dark infuscate, venation and stigma dark grey; haltere matte brown; scutal chaetotaxy (pairs): notopleural, 4; supra alar, 1; post alar, 1; dorsocentral, 4; scutellar, 1. *Abdomen*. Glossy black with short dark setae distributed evenly; erect whitish setae laterally on segments 1 and 2; segments 7 and 8 orange in colour with sparse, erect black setae. *Female genitalia*. Three spermathecae and a relatively small, simple spermathecal sac.

**Figure 5.**
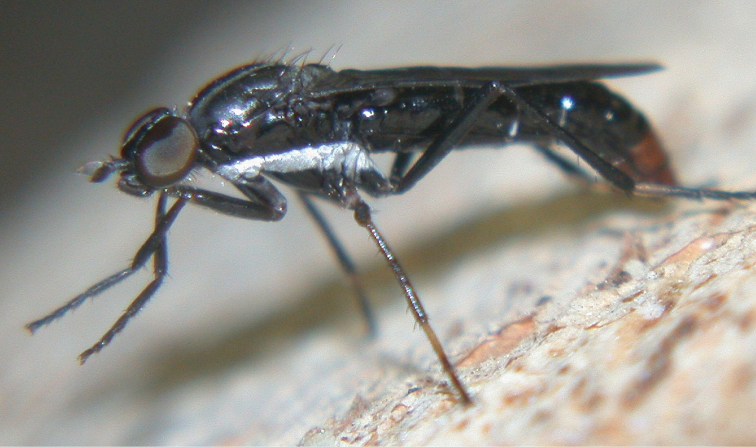
*Vomerina micora* sp. n., female habitus. Body length = 8.0 mm.

**Figure 6. F6:**
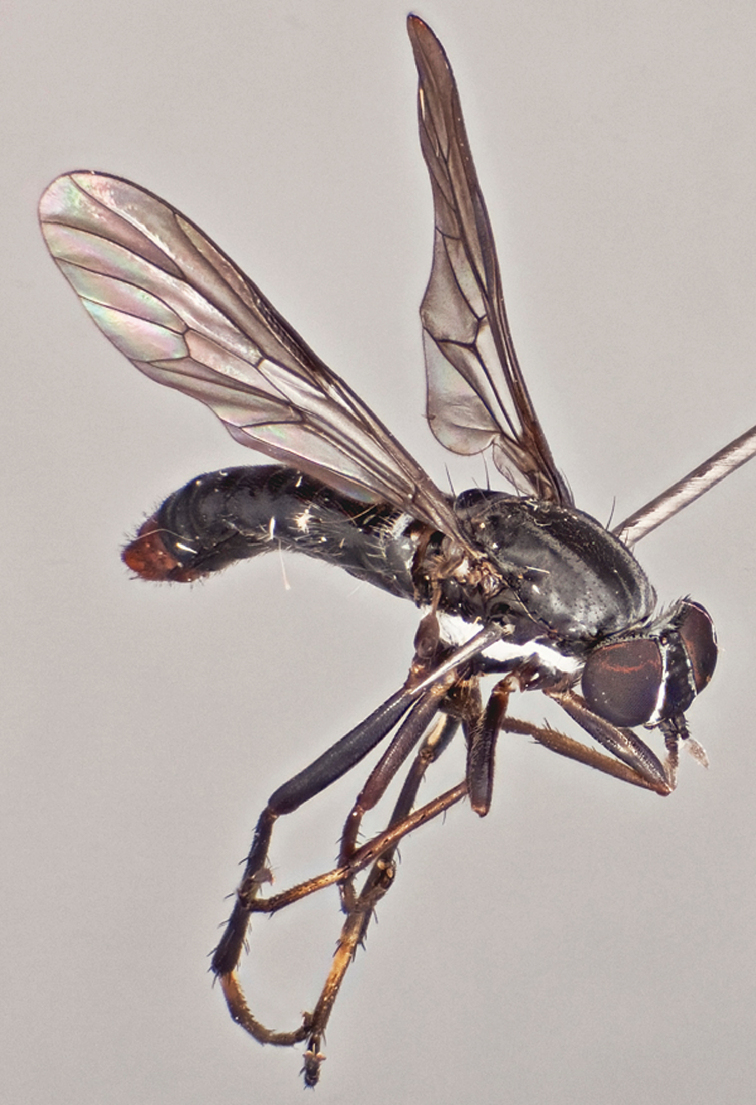
*Vomerina micora* sp. n., female habitus, oblique view. Body length = 8.0 mm.

**Figure 7.**
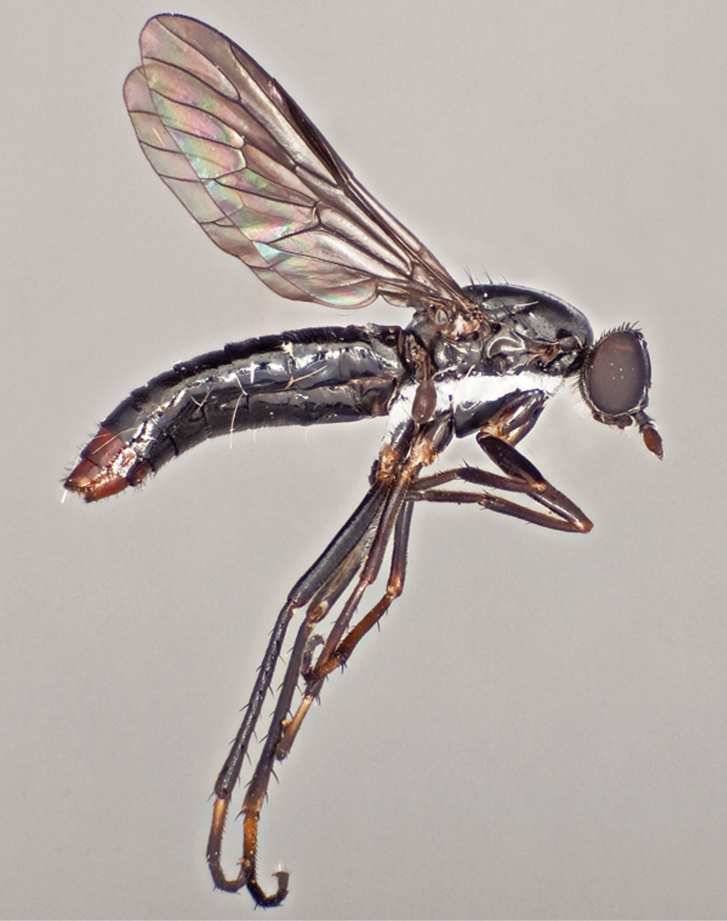
*Vomerina micora* sp. n., female habitus, lateral view. Body length = 8.0 mm.

#### Etymology.

The species epithet is derived from Latin, *mico* shine; *ora* border; reference to the silver-grey pubescence border of the eye.

#### Comments.

*Vomerina micora* sp. n. is more slender in body than *Vomerina humbug* and *Vomerina comapenis* sp. n. and can be distinguished by the dorsocentral stripes on the scutum, short, cylindrical scape, and the pleural stripe not extending onto the abdomen. The orange terminal abdominal segments may prove to be a sexually dimorphic character, as is found in many other therevid species. The male is unknown.

## Supplementary Material

XML Treatment for Vomerina

XML Treatment for Vomerina comapenis

XML Treatment for Vomerina micora
